# Challenges and opportunities for ELSI early career researchers

**DOI:** 10.1186/s12910-016-0121-5

**Published:** 2016-07-08

**Authors:** Jessica Bell, Mirko Ancillotti, Victoria Coathup, Sarah Coy, Tessel Rigter, Travis Tatum, Jasjote Grewal, Faruk Berat Akcesme, Jovana Brkić, Anida Causevic-Ramosevac, Goran Milovanovic, Marianna Nobile, Cristiana Pavlidis, Teresa Finlay, Jane Kaye, ELSI2.0

**Affiliations:** HeLEX - Centre for Health, Law and Emerging Technologies, Nuffield Department of Population Health, University of Oxford, Oxford, United Kingdom; Centre for Research Ethics & Bioethics, Uppsala University, Uppsala, Sweden; VU University Medical Center, Dept. of Clinical Genetics, Section of Community Genetics, EMGO Institute for Health and Care Research, Amsterdam, The Netherlands; National Institute for Public Health and the Environment (RIVM), Bilthoven, The Netherlands; Dynamics Lab - UCD Geary Institute for Public Policy, University College Dublin, Dublin, Ireland; CELLS - Centre for Ethics and Law in the Life Sciences, Leibniz Universitaet Hannover, Hannover, Germany; Genetics and Bioengineering, International University of Sarajevo, Sarajevo, Bosnia and Herzegovina; Centre for Developing Pharmaceutical and Biochemical Practice, Faculty of Pharmacy, University of Belgrade, Belgrade, Serbia; Bosnalijek Joint Stock Company, Pharmaceutical and Chemical Industry, Jukiceva 53, Sarajevo, Bosnia and Herzegovina; DiploFoundation, Belgrade, Serbia; Dipartimento di Giurisprudenza, Università degli Studi di Milano-Bicocca, Milan, Italy; University of Patras, Rio, Western Greece Greece; School of Social Sciences, Cardiff University, Cardiff, UK; The ELSI2.0 Collaboratory, https://elsi2workspace.tghn.org/

**Keywords:** Early career researcher, ELSI, Interdisciplinary, Web 2.0, Collaboration, Networks, Mentoring

## Abstract

**Background:**

Over the past 25 years, there has been growing recognition of the importance of studying the Ethical, Legal and Social Implications (ELSI) of genetic and genomic research. A large investment into ELSI research from the National Institutes of Health (NIH) Human Genomic Project budget in 1990 stimulated the growth of this emerging field; ELSI research has continued to develop and is starting to emerge as a field in its own right. The evolving subject matter of ELSI research continues to raise new research questions as well as prompt re-evaluation of earlier work and a growing number of scholars working in this area now identify themselves as ELSI scholars rather than with a particular discipline.

**Main text:**

Due to the international and interdisciplinary nature of ELSI research, scholars can often find themselves isolated from disciplinary or regionally situated support structures. We conducted a workshop with Early Career Researchers (ECRs) in Oxford, UK, and this paper discusses some of the particular challenges that were highlighted. While ELSI ECRs may face many of the universal challenges faced by ECRs, we argue that a number of challenges are either unique or exacerbated in the case of ELSI ECRs and discuss some of the reasons as to why this may be the case. We identify some of the most pressing issues for ELSI ECRs as: interdisciplinary angst and expertise, isolation from traditional support structures, limited resources and funding opportunities, and uncertainty regarding how research contributions will be measured. We discuss the potential opportunity to use web 2.0 technologies to transform academic support structures and address some of the challenges faced by ELSI ECRs, by helping to facilitate mentoring and support, access to resources and new accreditation metrics.

**Conclusion:**

As our field develops it is crucial for the ELSI community to continue looking forward to identify how emerging digital solutions can be used to facilitate the international and interdisciplinary research we perform, and to offer support for those embarking on, progressing through, and transitioning into an ELSI research career.

## Background

### Introduction

The importance of understanding and responding to the Ethical, Legal and Social Implications (ELSI) of genetics and genomics research was first formally recognised in 1990 during the initial assessment of the Human Genome Project (HGP) proposal. The United States Congress mandated that “not less than” 5 % of the NIH Human Genome Project budget would be set aside for research on the ethical, legal, and social implications of genomic science, establishing the ELSI Program of the National Institutes of Health (NIH) [1]. This was the largest ever investment in bioethics research [1] and stimulated the growth of an emerging field within the USA. Similar programmes have since been instigated in other countries, such as Canada, Australia and the UK.

In the 25 years since the HGP, ELSI research has continued to develop in step with its evolving subject matter and is starting to emerge as a field in its own right. The ELSI community has continued to produce work that plays an important role within society, leading to a number of policy changes [2]. ELSI researchers have regularly been partners within large scientific consortia where they have had to address and find solutions to some of the challenges raised by genome research as they have unfolded. However, the role and importance of ELSI research has not been uncontested. Questions have been raised over sources of funding and the potential influence on the research agenda [3], and whether ELSI research should be driven by public policy or the scientific community [4, 5]. Critics of ELSI have suggested that its research is not truly collaborative and contributions made by social scientists, lawyers and ethicists are ‘often limited to narrow, prescriptive positions’ [6]. Indeed, one of the greatest challenges addressed by ELSI research is the need to facilitate ethical genomic research while also providing critical assessment of advancements being made [7].

In response to fast moving scientific agendas, ELSI researchers have taken the tools and approaches from their foundational disciplines of science, bioethics, philosophy, law and sociology and applied them to the challenges in genomics. This has often resulted in new interdisciplinary methodologies and approaches as well as research that is firmly grounded in issues from scientific practice. As a result ELSI research often combines a strong theoretical foundation or approach with empirical evidence that is used to guide and inform future policy and best practice. While those contributing to this ancillary field originally came from a range of disciplines, individuals and groups undertaking ELSI research are increasingly identifying themselves as belonging to a new field, with its own distinctive approach. If we are to continue to develop our nascent community we must take time to reflect on the challenges faced by those who are new to the field, Early Career Researcher (ECRs)^1^ embarking on, or continuing, their careers as ELSI researchers.

In November 2014, the University of Oxford HeLEX Centre for Health, Law and Emerging Technologies and the European Cooperation in Science and Technology (COST) Action CHIP-ME^2^ held a workshop for ECRs, including ECRs from the ELSI community.^3^ The key aims of the workshop were to identify and discuss the issues faced by ECRs, including both those who identify themselves as ELSI scholars and their contemporaries from other fields, and to highlight and explore potential solutions using web 2.0 technology. First, this paper will discuss the challenges for ELSI ECRs highlighted during the workshop, which include: interdisciplinary angst and expertise; isolation from traditional support structures; limited resources and funding opportunities; and uncertainty around how research contributions will be measured. Second, the potential solutions using web 2.0 technology are outlined, and these include: increased accessibility to mentoring and peer support; enhanced networking opportunities; improved access to resources; and accreditation systems that reflect a broader array of contributions.

## Main text

### Interdisciplinary angst and expertise

Many of the challenges faced by ELSI ECRs are not dissimilar to those experienced by ECRs in other disciplines. ELSI ECRs are faced with the universal academic challenges of gaining funding, uncertain career prospects and struggling to gain standing or recognition in their discipline. In addition, scholars at all levels who are involved in interdisciplinary collaborations have to deal with geographic and disciplinary distances that make productive research more difficult to achieve. Interdisciplinary research, defined as the ‘production of research which crosses disciplinary boundaries’ [8], is a means of addressing complex problems that cannot be dealt with from a single disciplinary perspective; bringing together different approaches to work together, share ideas, theories and practice to reach appropriate solutions [9]. However, conditions must be created for effective cooperation and interdisciplinary research raises well-documented issues associated with collaboration across disciplinary silos. These include communication difficulties when crossing geographical time-zones, variable funding priorities and different teaching, research and reward practices between institutions [10].

Arguably, these challenges are amplified for ELSI researchers due to a number of reasons. First, ELSI is a new field of scholarship with rapidly emerging areas of novel enquiry. This may mean that the infrastructures observed in other long-standing, traditional disciplines, for example learned societies and associated journals, professional bodies and charitable organisations, are not as well-established. Second, ELSI research is typically ‘big interdisciplinary’ [11], which refers to collaborative work between distant disciplines, such as developmental biology and philosophy [12]. This may introduce structural problems (i.e. the physical distance between research centres) as well as difficulties presented by sociocultural and epistemological differences between disciplines [9]. Third, ELSI research is also commonly ‘trans-disciplinary’, meaning it brings together perspectives from a broad range of stakeholders, including non-academics, such as industry partners, advocacy groups, lay representatives and law and policy makers [13]. This may create challenges associated with navigating and including perspectives from a range of stakeholders, each with potentially different motives, expectations, sources of funding, and methods of working.

Furthermore, ELSI research must evolve and develop in reaction to rapidly advancing technology, which means ECRs may be one of very few researchers exploring a new line of investigation. ELSI early career researchers may be more likely to progress quickly to the status of ‘experts’ on the basis of their subject specific knowledge, which could be disproportionate to their wider expertise as researchers in the academic context. Rather than experiencing the positive benefits and opportunities afforded by rapidly advancing from a position of novice to expert, they may encounter expectations that they find demanding to fill. On the matter of expertise, Collins and Evans’ ‘Periodic Table of Expertise’ is helpful to illustrate some of the different conceptualisations of expertise: contributory experts are those who have in-depth knowledge and practical competence in their specialist area and are those known by others for their contributions to the field; whereas interactional experts are able to ‘talk the talk’ in a convincing manner in their specialist area, but would not be expected to practise competently in it [14]. Additional types and levels of capability support Collins and Evans’ assertions, but in this modest application of their theory we would argue that the unique issue for ELSI ECRs is that there is a potential gap or tension between their subject-specific ‘expertise’ and their wider expertise as researchers. It is this tension that may result in a lack of confidence in junior researchers in the performance of their various roles, which are not limited to application of subject specific knowledge, and which may give rise to unique challenges regarding others’ perceptions and expectations of junior researchers within their institution.

### Lack of support and isolation

Due to the global nature of ELSI research [7], ELSI ECRs may be geographically isolated from other ELSI scholars. ELSI ECRs are not only globally mobile; their affiliation between faculties and departments is also fluid and nomadic. For example, an ELSI researcher exploring the regulation of genetics could be based in faculties of Law, Medicine, Social Sciences or Human Sciences. ELSI ECRs may therefore be more likely to work in isolation without the benefits of the support, mentoring and networking opportunities that are usually associated with succession planning and career progression structures in conventional academic research groups [15]. While working alone can often provide more autonomy and the opportunity to think through issues individually, ELSI ECRs describe feelings of isolation similar to those previously reported in other disciplines [16]. Isolation appears to be particularly common amongst interdisciplinary ECRs, and in the case of ELSI ECRs this may be amplified by research being both ‘big interdisciplinary’ and ‘trans-disciplinary’. Subsequently, ELSI ECRs may find it difficult to integrate with their peers, without the common ground of their field of research.

Such isolation may impede one’s ability to develop the confidence that is necessary for career progression. In a study carried out by Hemmings and Kay, participants described how a lack of confidence negatively impacted on their research [17]. Evidence suggests that providing appropriate support and guidance will increase confidence [17] and result in enhanced networking, collaboration and productivity [18]. A number of studies have shown that networking can directly impact career progression, for instance by introducing opportunities for collaboration, funding and job vacancies, highlighting its importance to the development of ECRs [19–21]. Even in cases where ECRs have a solid range of knowledge and skills, individuals need confidence to apply these to their work [22] and to publish successfully [23].

Research has indicated that professionals in a transitional career stage, such as ECRs, often observe and emulate the behaviour of those in more senior roles [24]. For ECRs that straddle multiple disciplines, such as those engaged in ELSI research, identifying appropriate role models can be more difficult. Indeed, role models could be individuals from the private sector or industry partners, advocacy groups, law-makers, senior researchers and so on. While this may be seen as a challenge, ECRs with the confidence to engage and learn from a diversity of mentors arguably stand to gain unparalleled opportunities for development in terms of breadth of knowledge and networks, and thus suggestions as to how to harness these opportunities will be discussed later in this paper.

### Lack of funding and resources

Certain resources are crucial to the success of an ECR and arguably most important is access to funding. While this is not unique to ELSI ECRs, funding opportunities are especially complicated in this inherently interdisciplinary field because postdoctoral or other junior academic positions may span a number of faculties and disciplines. This may mean that ELSI ECRs don’t ‘fit the mould’ for many positions that are advertised. Keeping ECRs regularly informed of relevant funding and employment opportunities for interdisciplinary researchers is imperative for career progression because, fundamentally, the amount and source of funding has been associated with research performance [25]. A related issue is access to funding to cover the cost of journal subscriptions. Although access to online material is more widely available through open access policies, there are still many journals that require subscriptions, which can be prohibitive for ECRs in developing countries.

Beyond funding, additional barriers may still exist to access materials in other countries. The vast majority of scientific and legal literature is published in English, which gives advantage to those who are fluent speakers. In addition to this, ELSI ECRs may need to refer to literature that spans a wide range of disciplines, which may include unfamiliar jargon and acronyms. For example, an ELSI ECR with a background in genetics may find they need to learn about the regulatory frameworks for biobanks.

### Measuring contributions

The changing landscape of communication in academia has meant that many ECRs are also presenting their work through more informal channels, such as blogs, seminars and interest groups [26]. These can provide rich learning experiences, but they are not always recognized in the same way that journal articles or teaching awards may be. While LinkedIn and other online CV builders provide a platform for researchers to detail their achievements, the site is considered a formal and professional tool, and ECRs may not feel it is appropriate to list less widely recognized accomplishments on such a platform.

### Harnessing solutions to ELSI ECR challenges and opportunities

In this increasingly collaborative, competitive and mobile environment, it is more crucial than ever that ELSI ECRs have the tools they need to maximize global opportunities. ELSI ECRs need tailored opportunities, guidance and support to gain experience and develop professional networks to develop their careers and prepare to become the next generation of established ELSI scholars.

Mentoring is a traditional approach to support junior and senior academics in their career progression, however, one-on-one mentorship opportunities often do not extend beyond the PhD student/supervisor relationship. Post-doctoral researchers together with graduate students have often expressed the need for effective mentorship to progress beyond thesis submission [27], to progress confidently in their careers. For ELSI ECRs, working between distant disciplines may mean that there is little in the way of formal or traditional structures for mentoring which may be experienced in single discipline or established interdisciplinary institutions.

Digital approaches employing web 2.0 technology could offer those operating in a remote setting, the opportunity to meet, share stories and help other, which has been shown to be mutually beneficial [28, 29]. An online platform that offers mentorship could assist individuals in finding the support they require at any stage in their career, helping to build confidence in their work.

Beyond traditional formats such as seminars and project meetings, digital technologies could be used for new approaches to supporting researchers, such as ‘Ask the Expert’ webinar sessions, whereby a senior academic could give a short presentation on a given topic and respond to user submitted questions relating to their research. For ELSI ECRs who struggle to connect with peers at other institutions this could help offer a low cost means to forge networks and enable future collaboration. Discussion forums can provide an informal way for ECRs to initiate discussions with or seek advice from peers and more senior colleagues, whilst more broadly enabling the identification of fellow researchers with similar interests potentially stimulating new collaboration.

As well as permitting connectivity between remote researchers, web 2.0 approaches could be used to overcome the time zone limitations experienced by researchers operating in different regions. Transcripts or recordings of previous meetings can create a lasting resource, and discussion forums can provide an informal way for ECRs to initiate discussions with, or seek advice from, peers and more senior colleagues. Teleconferencing and webinars enable researchers to present aspects of their work to others within the ELSI field without requiring the resources for traveling great distances. This is not to undermine the importance of face-to-face meetings, and in addition it will often be necessary to attend events in person to build and maintain networks. Therefore, it remains important that ECRs have access to sustainable funding and resources to facilitate such mobility. If ECRs are able to connect with peers at other institutions, this could also help to build networks for future collaborations.

To facilitate web 2.0 connectivity in the ELSI arena, a centralized space could be developed where a number of different resources could be made available, including funding opportunities, access to articles, a translation tool and short guides to common terms and jargon used in specific fields. A number of websites already provide some of these services, such as Research Gate, LinkedIn, and Google translate. However, discrete logins and web addresses make them cumbersome to use on a daily basis and such duplication could be avoided by a centralized web-platform. Resources made available to the community on this space could include user and institutional profiles, details of funding opportunities and upcoming events, and resources such as publications, research tools, and contextual guides such as bibliographies of common terms and abbreviations relevant to specific fields of research.

Finally, in our emerging field, we have the opportunity to develop and encourage the use of new achievement metrics so that they become integral to the work that we perform. One way to achieve this would be to create a reward system that is based on community participation and which rewards participation through an accreditation system linked to individual user accounts of an integrated e-platform. Metrics contributing towards accreditation could incorporate conventional recognition systems such as academic activity and publication record, together with contemporary metrics that record an individual’s participation in user groups and publication through social media streams such as blog articles, thus recognizing the breadth of contributions made by the modern day researcher. Similar recognition systems adopted by other fields include the ResearchGate impact factor and the Bioresource Research Impact Factor for those operating in the field of biobanks [30].

## Conclusions and future solutions

Web 2.0 technologies offer the potential to revolutionize academic support structures as well as the research they enable. We see the potential for a new approach to academic networking and collaboration that exists in the digital landscape, employing digital tools to facilitate collaborative activity amongst ELSI scholars; this is mirrored by a broader desire to build a centralized e-workspace for academia as a whole [31]. However, whilst digital solutions exist for many of the issues facing ELSI ECRs, we are not aware of any unified offering that could address them all.

Over the year following the Oxford workshop, ELSI ECRs worked with ELSI2.0 to identify how it can use current technologies to facilitate international networking, discussion and collaboration relating to ELSI research. This free and open network for ELSI researchers offers a website where researchers can learn about ELSI meetings and activities taking place around the world, or publicize their own event; find links to ELSI research groups and organizations and add their own affiliations; and learn about opportunities to become involved in network activity. To address the need for global interactions between ELSI researchers, the website provides information about topic, activity or community specific ‘Making Connections’ groups, through which members can get in touch with researchers with whom they identify shared interests, or create their own group to stimulate new collaborative activity.

In a digitally connected world we are discovering new tools that can be used to support researchers and through which members can freely and instantaneously communicate amongst themselves and share resources. Similar practice has also emerged among groups in healthcare management sectors [32] and we hope that our suggestions for web 2.0 solutions can be applied more broadly, across various disciplines and sectors. For those limited by resources, including those who are studying, early in their career, or with low economic means, it is hoped that these connections will go some way to helping level the playing field between those with and without strong institutional support, as well as directing resources to where they are needed more rapidly.

As our field develops it is crucial for the ELSI community to continue looking forward to identify how emerging digital solutions can be used to facilitate the international and interdisciplinary research we perform and to offer support for those embarking on, progressing through, and transitioning into an ELSI research career. This is also essential if we want to further ELSI research as a field that is not defined just by its sphere of investigation, but by its approach, methodologies and the quality of the training and support that it gives to its future leaders – the ECRs.

## Abbreviations

COST, European Cooperation in Science and Technology; ECR, Early career researcher; ELSI, Ethical legal and social implications; ELSI2.0, An international collaboratory for genomics and society research (https://elsi2workspace.tghn.org/); HGP, Human genomes project; NIH, National Institutes of Health (USA)

## Footnotes

1 Early Career Researcher (ECR) defined as Doctoral Students or Researchers who completed their PhD within the past 10 years and do not hold a permanent position.

2 Citizen’s Health through public-private Initiatives: Public health, Market and Ethical perspectives (CHIP ME).

3 The workshop was held in Oxford, UK, across five days. Thematic presentations, breakout sessions and social activities were conducted to encourage in depth discussions about the challenges and potential solutions. Participants included 16 ECRs (10 Doctoral Students) based in nine different European countries. No raw data was collected from the workshop.
